# The predictive value of serum S100A9 and response to etanercept is not confirmed in a large UK rheumatoid arthritis cohort

**DOI:** 10.1093/rheumatology/kew387

**Published:** 2017-01-16

**Authors:** Samantha Louise Smith, Darren Plant, Stephen Eyre, Kimme Hyrich, Ann W. Morgan, Anthony G. Wilson, John D. Isaacs, Anne Barton

**Affiliations:** 1 Arthritis Research UK, Centre for Genetics and Genomics, Centre for Musculoskeletal Research, Division of Musculoskeletal and Dermatological Sciences, Faculty of Biology, Medicine and Health, Manchester Academic Health Sciences Centre, The University of Manchester; 2 National Institute for Health Research Manchester Musculoskeletal Biomedical Research Unit, Central Manchester Foundation Trust and University of Manchester, Manchester Academic Health Science Centre; 3 Arthritis Research UK, Centre for Epidemiology, Centre for Musculoskeletal Research, Division of Musculoskeletal and Dermatological Sciences, Faculty of Biology, Medicine and Health, Manchester Academic Health Sciences Centre, The University of Manchester; 4 Leeds Institute of Rheumatic and Musculoskeletal Medicine, University of Leeds and National Institute of Health Research Leeds Musculoskeletal Biomedical Research Unit, Leeds Teaching Hospitals National Health Service Trust, Leeds, UK; 5 University College Dublin School of Medicine and Medical Science, Conway Institute, University College Dublin, Dublin, Ireland; 6 Musculoskeletal Research Group, Institute of Cellular Medicine, Newcastle University and National Institute of Health Research Newcastle Biomedical Research Centre, Newcastle upon Tyne National Health Service Foundation Trust, Newcastle upon Tyne, UK

**Keywords:** rheumatoid arthritis, biomarker, treatment response, protein, precision medicine, etanercept

## Abstract

**Objective.** The aim was to correlate protein concentrations of S100A9 in pretreatment serum samples with response to the tumour-necrosis factor (TNF) inhibitor drugs etanercept in a large UK replication cohort.

**Methods.** Pretreatment serum samples from patients with RA (n = 236) about to commence treatment with etanercept had S100A9 serum concentration measured using an ELISA. Following the experimental procedure, S100A9 concentrations were analysed with respect to EULAR response.

**Results.** No evidence of association between S100A9 concentration and EULAR response to the TNF-inhibitor biologic drug etanercept was observed following multinomial logistic regression analysis (non-responder *vs* moderate responder, P = 0.957; and non-responder *vs* good responder, P = 0.316). Furthermore, no significant associations were observed when correlating pretreatment S100A9 concentrations with clinical parameters of disease activity (P > 0.05).

**Conclusion.** In the largest replication cohort conducted to date, no evidence for association was observed to support the use of S100A9 as a clinical biomarker predictive of response to the TNF-inhibitor biologic drug etanercept.

Rheumatology key messages

- This is the first study to attempt replication of the S100A9 protein biomarker in a large Caucasian RA cohort.
- The S100A9 protein biomarker is not predictive of EULAR response to etanercept in RA.

## Introduction

Biologics have revolutionized the treatment of RA, greatly benefitting the majority of patients receiving them. It is known that early and effective treatment is key in order to minimize joint damage [1, 2], but biologics are prescribed on what is essentially a trial-and-error basis, and effective treatment is not always achieved. In fact, up to 30–40% of patients on biologics fail to respond satisfactorily, and the disease can continue to progress, potentially resulting in increased disability. To date, a reliable biomarker predictive of response to biologics has yet to be identified despite being the aim of precision medicine initiatives. The necessity for such a biomarker was re-emphasized by a study which demonstrated that patients may continue their current regime despite an inadequate response for far >6 months [3], which is the time point at which the National Institute for Health and Care Excellence recommend switching to an alternative biologic [4].

The S100 protein family could provide one such promising biomarker. These multifunctional proteins have been found to be upregulated in inflammatory disorders, including RA [5–7], levels commonly correlate with clinical markers of disease activity (such as ESR and CRP) [8–13], and they have been found to be suppressed (both locally at the site of inflammation and distally within the circulatory system) following treatment with biologics [8, 10, 13–15]. In fact, a recent study has investigated the predictive value of S100A8, S100A9 and S100A8/A9 in pretreatment serum samples collected from RA patients (n = 22), using both mass spectrometry (relative quantification) and ELISAs (absolute quantification). It was demonstrated that increased levels of S100A9 before treatment (using both relative and absolute quantification) were predictive of response to the TNF-inhibitor biologic drug etanercept (P = 0.023) [16]; however, no significant differences were observed when correlating absolute levels of S100A8 and S100A8/A9 with the response phenotypes. It is important that replication of the S100A9 association be attempted in independent cohorts in order to confirm the correlation with treatment response. The aim of this research was, therefore, to replicate the association with S100A9 in a larger cohort of UK RA patients about to commence treatment with etanercept.

## Methods

### Patient selection

Patients with RA were selected from the Biologics Prospective Study, the prospective arm of the Biologics in RA Genetics and Genomics Study Syndicate (BRAGGSS), which recruits patients who are about to commence treatment with biologic drugs from >50 sites across the UK, described in detail previously [17]. Patients provide blood samples and psychological and clinical information. This is repeated after 3, 6 and 12 months. As such, disease activity in 28-joints (DAS28) scores using four variables (the number of tender and swollen joints, ESR/CRP and patient global assessment score) can be calculated before and after treatment [18]. The BRAGGSS study was approved by National Research Ethics Service Committee North West—Greater Manchester South (Research Ethics Committee Ref: 04/Q1403/37). This approval included the present study, so no additional approval was required for this study.

Inclusion criteria for this study were as follows. Participants were: Caucasian; >18 years old; fulfilled the 1987 ACR criteria for RA; gave written informed consent; were about to commence treatment with the biologic drug etanercept; and had an available pretreatment serum sample for analysis.

### Defining treatment response

Clinical effectiveness was assessed using the EULAR classification criteria [19]. A good response was defined as a follow-up DAS28 joints of ⩽3.2 and having decreased from the pretreatment DAS28 score by >1.2. A non-response was defined as having a DAS28 score that decreased <0.6 from the pretreatment DAS28 score or decreased between 0.6 and 1.2 but having an end score of >5.1. Moderate response was classified when responses fell intermediate to these two extremes.

### Serum collection

Upon receipt, blood samples (previously collected into plain blood tubes) were centrifuged at 1845 RCF for 10 min. After centrifugation, the serum was aliquotted and stored at −80 °C until required.

### ELISA

Concentrations of S100A9 were determined in pretreatment serum samples using an ELISA according to the manufacturer’s instructions (Cusabio®, Hubei Province, China). The detection range for the assay was 4.69–00 ng/ml; if a concentration outside this range was recorded, the sample was diluted and the assay repeated. Likewise, if duplicate samples differed by >20%, the assay was repeated. Absorbance was measured using the SpectraMax Plus^384^ Absorbance Microplate Reader (Molecular Devices, CA, USA), with 450 nm as the primary wavelength and 540 nm for wavelength correction. All samples and standards were assayed in duplicate.

### Statistical analysis

All analyses were conducted in STATA/SE v11.2 [20]. Kruskal–Wallis rank sum and analysis of variance tests were used to assess the relationship between baseline clinical/demographic data and EULAR response. Multinomial logistic regression was conducted to determine the relationship between pretreatment S100A9 concentrations and EULAR response, using non-response as the base outcome. Covariates, in terms of the baseline characteristics (i.e. age at baseline) were added to the model if statistically different between the response phenotypes. Spearman rank correlations were conducted to determine the relationship between pretreatment S100A9 concentrations and clinical parameters (i.e. CRP and pretreatment DAS28 scores). A value of P < 0.05 was considered statistically significant for all analyses. Power calculations were performed using G*Power version 3.1.2 [21].

## Results

Two hundred and fifty-four pretreatment serum samples were available for analysis. Owing to limited reagents, 18 samples were excluded because the duplicate samples differed by >20% or the concentration was outside the range of the standard curve; this left 236 samples for analysis. Using the EULAR classification criteria, this equated to 44 non-responders, 98 moderate responders and 94 good responders. For the majority of the samples, the response was assessed at 3 months (n = 200), whereas for the remainder, the response was assessed at 6 months (n = 36) whenever response could not be calculated at 3 months. Cohort characteristics for these 236 samples are listed in Table 1.

**Table 1:** Baseline characteristics of the 236 serum samples analysed for S100A9

Cohort characteristics	Non-responders (n = 44)	Moderate responders (n = 98)	Good responders (n = 94)	P-value
Gender, female, n (%)	39 (88.6)	78 (79.6)	73 (77.7)	0.303^a^
Age at baseline, mean (s.d.), years	58.3 (11.9)	58.9 (11.5)	55.4 (11.7)	0.111^b^
Concurrent DMARDs, n (%)	42 (95.5)	70 (71.4)	82 (87.2)	<0.001^a^
DAS28 score at baseline, median (IQR)	5.6 (5.0–6.3)	6.1 (5.5–6.6)	5.8 (5.1–6.2)	0.0032^c^
DAS28 score at outcome, mean (s.d.)	5.3 (4.9–5.7)	4.2 (3.6–4.6)	2.4 (1.8–2.8)	<0.001^b^
Change in DAS28 score, mean (s.d.)	−0.18 (0.66)	−1.9 (0.86)	−3.5 (1.00)	<0.001^b^
TJC, median (IQR)	14 (10–20)	16 (11–22)	13.5 (9–19)	0.047^c^
SJC, median (IQR)	8 (3–11)	9 (6–12)	9 (6–13)	0.176^c^
CRP, median (IQR)	9.3 (3.5–21.7)	15 (7.3–31.2)	10 (3.5–27.2)	0.106^c^
HAQ, median (IQR), n	2 (1.3–2.4), 25	1.8 (1.3–2.1), 67	1.6 (1.3–2), 66	0.152^c^

a Calculated using χ^2^ test. ^b^Calculated using one-way analysis of variance. ^c^Calculated using Kruskal–Wallis test. DAS28: disease activity scores in 28-joints ; IQR: interquartile range; SJC: swollen joint count; TJC: tender joint count.

**Fig. 1:**
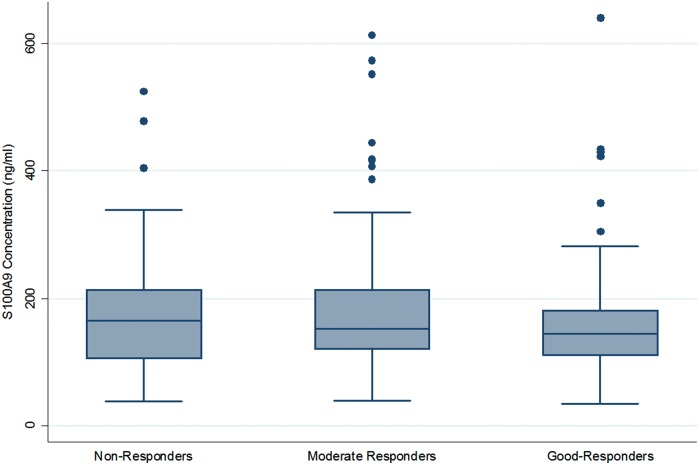
Pretreatment serum S100A9 concentrations grouped according to EULAR response phenotypes Absolute quantification of S100A9 from baseline serum samples in non-responders, moderate responders and good responders assessed by sandwich ELISA. The upper and lower quartiles are represented as the upper and lower bounds of the box, whilst the line within the box represents the median. The whiskers represent the minimal and maximal values. Outliers are represented as small dots.

Mean (s.d.) S100A9 concentrations for the three EULAR response phenotypes were 183.8 (109.6) ng/ml in non-responders, 183.7 (109.5) ng/ml in moderate responders and 165.5 (91.7) ng/ml in good responders. Using non-response as the base outcome, multinomial logistic regression was used to determine the association between pretreatment S100A9 concentrations and EULAR response, with concurrent DMARD use and baseline DAS28 score as covariates. This resulted in non-significant associations [non-responder *vs* moderate responder, P = 0.957, odds ratio, OR (95% confidence interval (CI): 1.0 (0.997, 1.003); and non-responder *vs* good responder, P = 0.316, OR (95% CI): 0.998 (0.995, 1.002); Fig. 1]. Multivariate logistic regression comparing moderate and good responders gave a P-value of 0.225, OR (95% CI): 0.998 (0.995, 1.001). Additionally, using a multivariate logistic regression model, the analysis was repeated after the grouping of moderate and good responders into a single responder phenotype [P = 0.574, OR (95% CI): 0.999 (0.996, 1.002)].

As the previously reported association was observed in patients receiving a combination of etanercept and MTX, the analysis was repeated after the exclusion of patients not receiving concurrent DMARDs (n = 42); however, this did not significantly alter the findings [non-responder *vs* moderate responder, P = 0.763, OR (95% CI): 1.00 (0.997, 1.004); and non-responder *vs* good responder, P = 0.206, OR (95% CI): 0.998 (0.994, 1.001)].

Further analysis was conducted to investigate whether there was a correlation between pretreatment S100A9 concentrations in serum and clinical baseline parameters, as it has previously been demonstrated that pretreatment concentrations of the family of S100 proteins (namely S100A8/A9) correlate with clinical markers of disease activity such as DAS28 scores (baseline and outcome), CRP and ESR [8, 10, 13–15]; however, no significant correlations were observed within this data set (P > 0.05).

DAS28 scores were also available at 6 months for 173 of these patients, in line with the previously reported study outcome assessment time point [16]; however, after the exclusion of moderate responders and those not on concurrent DMARD treatment, this equated to 90 patients available for further analysis at 6 months (22 non-responders and 68 responders). Multivariate logistic regression between these two phenotypes yielded a non-significant association [P = 0.353, OR (95% CI): 0.997 (0.993, 1.003)].

## Discussion

It has previously been reported that pretreatment serum concentrations of the protein S100A9 correlate significantly with response to treatment with the TNF-inhibitor biologic drug etanercept (P = 0.023) [16]. That was the first report of a pretreatment protein biomarker successfully correlating with response to etanercept and, therefore, it is important to replicate the association. However, in contrast to the previous study, in the present large, well-powered replication cohort, we found no evidence to support the use of pretreatment serum S100A9 concentrations as a predictor of response to etanercept (non-responders *vs* moderate responders, P = 0.957; and non-responders *vs* good responders, P = 0.316).

Furthermore, we found no statistically significant association with DAS28 at follow-up (when relative quantification using mass spectrometry was found to be correlated with 6 month DAS28 scores within the original study, P = 0.016 [16]) or with baseline DAS28, CRP, swollen joint count or tender joint count, which is again in contrast to previous studies investigating other members of the S100 family [8, 9, 13].

A strength of the present study was the large sample size tested (n = 236) in comparison with the original cohort (n = 22), reducing the chance of a false-negative result. For example, using G*Power 3.1.2, in order to achieve 80% power to detect moderate to large differences in protein abundance between the response phenotypes at a significance level (α) of 0.05 and using a one-tailed distribution, 31 non-responders and 125 responders would have been needed. However, there are several possible reasons for the lack of replication. First, moderate responders were included within the present analysis as compared with the original study where they were excluded. However, following multinomial logistic regression, comparing non-responders with good responders, no evidence for association was detected (P = 0.316). In fact, moderate responders demonstrated the most similar S100A9 signature to non-responders, suggesting that the inclusion of moderate responders was not a confounding issue. Second, a major difference between the two studies is that the response to etanercept was predominantly assessed at 3 months in the present study as compared with the 6 month time point in the original report. However, further investigation following stratification by the assessment time point did not materially alter the conclusions (data not shown).

Another reason for the lack of replication could be attributable to the fact that the previously reported study also used mass spectrometry to assess further the relative abundances of S100A9 protein peptides within patient serum samples. The authors reported that responder patients overexpress three protein peptides that are not expressed in non-responders and, subsequently, accounted for this within their analyses by normalizing across the data set; this normalization step could therefore explain the discordant association. However, the technology was not available for use in the present study, and if, indeed, the S100A9 association is dependent upon normalization for peptide abundances, it seems unlikely that this biomarker would be readily adopted for routine use in clinics. Furthermore, the original study detected only a modest association of S100A9 as a biomarker for response to etanercept, with sensitivity and specificities that are considered too low to be adopted for widespread implementation (Receiver operating characteristics sensitivity, 83%; specificity, 70%).

Another consideration that needs to be taken into account is the way in which serum samples are collected and processed. As part of the BRAGGSS cohort, blood samples for serum collection are shipped via postal services, resulting in a median lag time of 1–5 days before the blood sample is received and processed. This is an important factor to take into account because proteins may be prone to degradation during this period at room temperature and this may explain the lack of replication between the present study and the previously reported association. As far as can be determined, S100 protein stability has been investigated in serum samples only for S100A12. It was demonstrated that for freshly drawn blood left at room temperature for up to 48 h before separation, the stability was highly dependent upon the tube into which the blood was drawn. For example, blood drawn into empty tubes, EDTA tubes or heparin tubes demonstrated increased S100A12 concentrations over time, whereas blood drawn into serum gel tubes demonstrated stable concentrations [22]. Furthermore, up to 10 freeze–thaw cycles did not significantly impact upon serum S100A12 concentrations at −20 °C (P = 0.26) or −70 °C (P = 0.30), and serum concentrations were stable for 6 months at −20 °C [22]. The delay between blood draw and sample processing was considered as a potential confounder in the present study. However, following multinomial logistic regression to compare S100A9 concentrations using a lag time of 1 day as the base comparison, no significant associations (P < 0.05) were observed (data not shown). This suggests that S100A9 is stable at room temperature for extended periods and was not therefore the reason for the discordant association between the present study and the previously reported observation.

Of note, the protein family of S100 proteins have consistently been reported to decrease significantly following treatment with biologics and, in some cases, this was significant following a mere 4 weeks of treatment [8, 10, 13–15]; however, S100A9 has not yet been tested to our knowledge. It could be that S100A9 is an early pharmacodynamic marker of response, but this will require further investigation in follow-up serum samples.

## Conclusion

In summary, the present study has conducted the largest replication cohort to date for S100A9, but no evidence for association with response to the TNF-inhibitor biologic etanercept or with subcomponents to the DAS28 score was observed.
